# Implementation Outcomes of Reusable Learning Objects in Health Care Education Across Three Malaysian Universities: Evaluation Using the RE-AIM Framework

**DOI:** 10.2196/63882

**Published:** 2025-07-23

**Authors:** Hooi Min Lim, Chin Hai Teo, Yew Kong Lee, Ping Yein Lee, Kuhan Krishnan, Zahiruddin Fitri Abu Hassan, Phelim Voon Chen Yong, Wei Hsum Yap, Renukha Sellappans, Enna Ayub, Nurhanim Hassan, Sazlina Shariff Ghazali, Nurul Amelina Nasharuddin, Puteri Shanaz Jahn Kassim, Faridah Idris, Klas Karlgren, Natalia Stathakarou, Petter Mordt, Stathis Konstantinidis, Michael Taylor, Cherry Poussa, Heather Wharrad, Chirk Jenn Ng

**Affiliations:** 1 Department of Primary Care Medicine, Faculty of Medicine, Universiti Malaya, Lembah Pantai, Kuala Lumpur, 50603, Malaysia, 60 379492621; 2 UMeHealth Unit, Faculty of Medicine, Universiti Malaya, Kuala Lumpur, Malaysia; 3 Dean's Office, Faculty of Medicine, Universiti Malaya, Kuala Lumpur, Malaysia; 4 Department of Building Surveying, Faculty of Built Environment, Universiti Malaya, Kuala Lumpur, Malaysia; 5 School of Biosciences, Faculty of Health & Medical Sciences, Taylor's University, Selangor, Malaysia; 6 School of Pharmacy, Faculty of Health & Medical Sciences, Taylor's University, Selangor, Malaysia; 7 Taylor's Digital, Taylor's University, Selangor, Malaysia; 8 Learning Innovation and Development, Centre for Future Learning, Taylor’s University, Selangor, Malaysia; 9 Department of Family Medicine, Faculty of Medicine and Health Sciences, Universiti Putra Malaysia, Serdang, Malaysia; 10 Department of Multimedia, Faculty of Computer Science and Information Technology, Universiti Putra Malaysia, Selangor, Malaysia; 11 Department of Pathology, Faculty of Medicine and Health Sciences, Universiti Putra Malaysia, Selangor, Malaysia; 12 Department of Learning, Informatics, Management and Ethics, Karolinska Institutet, Stockholm, Sweden; 13 NettOp, Department of E-Learning Development, University of Stavanger, Stavanger, Norway; 14 School of Health Sciences, University of Nottingham, Nottingham, United Kingdom; 15 Centre for Population Health Research and Implementation, SingHealth Regional Health System, Singapore, Singapore; 16 Duke-NUS Medical School, Singapore, Singapore

**Keywords:** e-learning, RE-AIM, implementation, dissemination, reusable learning objects, medical education, reach, effectiveness, adoption, maintenance

## Abstract

**Abstract:**

**Background:**

Current e- learning evaluation focuses on learners’ knowledge gain, satisfaction, perceptions, and attitudes; few assess the implementation outcomes of e- learning resources in teaching and learning.

**Objective:**

In this study, we used the RE-AIM (Reach, Effectiveness, Adoption, Implementation, and Maintenance) framework to systematically evaluate the implementation outcomes of reusable learning objects (RLOs) in the context of health care education.

**Methods:**

This study is a part of the Advancing Co-creation of RLOs to Digitise H ealthcare Curriculum (ACoRD) project, wherein we developed and implemented 23 RLOs across 3 Malaysian universities for medical, pharmacy, and biomedical curricula. Implementation and dissemination strategies were employed. Data were collected using a self-administered web-based questionnaire and Google Analytics.

**Results:**

Th is study report s a cumulative RLO access of 7622 users from 48 countries ( reach). Users rated RLOs as very helpful ( 1452/ 2071, 70.1%) or helpful ( 601/ 2071, 29.1 %). Pre assessments and postassessments showed a significant improvement in the knowledge score (21 RLOs, *P* < .05) and confidence level (17 RLOs, *P* < .05) (e ffectiveness). All 3 Malaysian universities adopted RLOs in the fields of professional development, primary care medicine, medicine, pediatrics, nursing, pharmacy, and biomedicine ( adoption). The percentage of users who completed RLOs ranged from 5.6% ( 10/ 179) to 8 5% ( 78/ 92), with nonbounced users (users who viewed more than one page) ranging from 16.3% ( 165/ 1014) to 8 8.5 % ( 3 70/ 4 18) ( implementation). In the 4 months following the completion of the AC oRD project, a total of 2107 users accessed RLOs ( maintenance).

**Conclusions:**

We systematically evaluated the implementation of e- learning resources by using the RE-AIM framework, informing future strategies to integrate e- learning innovations in real-world teaching and learning practices.

## Introduction

The use of technology in medical education and health sciences is on the rise, aiming to enhance the knowledge, skills, and practice of medical students. e- Learning, the process of acquisition and use of knowledge distributed and facilitated by electronic means [1], has been shown to be effective in not only improving knowledge but also increasing students’ satisfaction in medical education [2,3]. The application of e- learning objects in medical education promotes self-directed and personali zed learning, serving as a complement to the conventional didactic teaching method in medical schools [4]. An e- learning object refers to a collection of digital materials structured in a meaningful way, aligned with a specific learning objective, and designed as independent, self-contained units of instructional materials [4].

Although institutions are devoting substantial resources to the development of e- learning, successful implementation of e- learning remains challenging and is often not systematically measured [5]. A systematic review showed that the evaluation of e- learning tends to focus on the assessment of learners’ knowledge, satisfaction, perceptions, and attitudes , and there is a lack of rigorous evaluation of its implementation outcomes in the real-world environment [6]. Implementation outcomes are the effects of deliberate and purposive action to implement a new intervention or practice [7]. In the context of e- learning, implementation outcomes include the acceptability, adoption, appropriateness, feasibility, fidelity, and sustainability of e- learning resources in teaching and learning [8]. When e- learning objects are released as open content, reach and discoverability become important.

To date, several models have been used to evaluate the effectiveness and implementation of e- learning resources. The Kirkpatrick model, an outcome-focused model, has been widely employed to evaluate the effectiveness of an educational program across 4 levels: reaction, learning, behavior, and results [9]; however, it does not evaluate the implementation outcomes. The Context-Input-Process-Product model [10] is designed to evaluate both the process and product to determine the success of an educational program. Nevertheless, this model primarily focuses on defining the contextual factors to improve performance [11]. The Analysis, Design, Development, Implementation, and Evaluation model [12], an instructional design model, offers a structured approach to guide the development and implementation of educational programs but falls short of providing metrics for measuring implementation. Therefore, there is a need for an e- learning implementation outcome framework that provides a comprehensive and objective evaluation of the implementation of e- learning resources.

RE-AIM (Reach, Effectiveness, Adoption, Implementation, and Maintenance) is an established implementation science framework used in the health care setting to measure how effectively an evidence-based intervention is implemented [13]. It has been used in several studies to evaluate the implementation outcomes of health care provider education programs [14,15]. In this study, we aimed to use the RE-AIM framework to evaluate the implementation outcomes of reusable learning objects (RLOs) , which are bite -sized, stand-alone interactive web-based resources with multimedia that focus on a single learning objective [16]. The findings from this study would provide evidence on the feasibility of using the RE-AIM framework in the context of health care education, describe the implementation strategies used in this project, and propose recommendations to improve the evaluation of e- learning resource implementation in higher educational institutions .

## Methods

### Study Design

**Figure 1.**
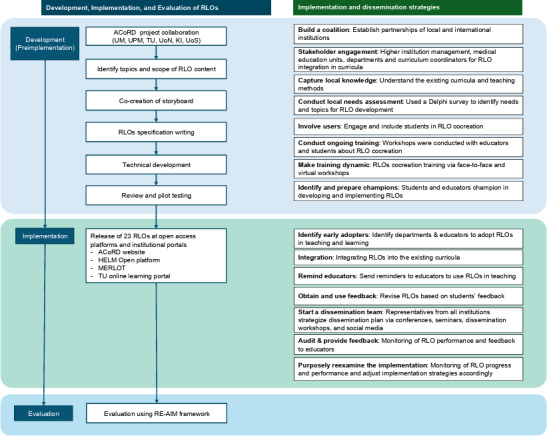
Development, implementation, and evaluation of reusable learning objects in the ACoRD project. ACoRD: Advancing Co-creation of Reusable Learning Objects to Digitise Healthcare Curriculum; HELM: Health e-Learning and Media; KI: Karolinska Institutet; MERLOT: Multimedia Educational Resource for Learning and Online Teaching; RE-AIM: Reach, Effectiveness, Adoption, Implementation, and Maintenance; RLO: reusable learning object; TU: Taylor’s University; UM: Universiti Malaya; UoN: University of Nottingham; UoS: University of Stavanger; UPM: Universiti Putra Malaysia.

This study is a part of the Advancing Co-creation of RLOs to Digitise H ealthcare Curricul um (ACoRD) project, which was funded by the European Union E rasmus+ project [17]. It is a capacity building project involving Universiti Malaya , Universiti Putra Malaysia , Taylor’s University , University of Nottingham, Karolinska Institutet, and University of Stavanger. The ACoRD project aimed to introduce innovative digital pedagogy methods by developing, evaluating, and disseminating high-quality, peer-reviewed RLOs that benefit health care and biomedical science learners in Malaysia. RLOs were developed, implemented, and evaluated over 3 phases (Figure 1). The implementation strategies of RLOs were developed and performed in both the development (preimplementation phase) and implementation phases.

### RLO Development Phase (Pre implementation)

RLOs were developed following the ASPIRE (Aim, Storyboarding, Populating, Implementing, Release, and Evaluation) framework [18]. The development phase began by establishing a coalition between international institutions within the project. We evaluated the process and challenges of transnational partnerships and knowledge transfer by using a qualitative study, providing insights on guiding effective partnership in e- learning development in the future [17]. This was followed by stakeholder engagement at 3 Malaysian institutions, involving higher education institution management, medical education units, respective departments, and curriculum coordinators. To identify the gaps and topics for RLO development, the ACoRD team reviewed the existing curricula and determined how RLOs can be integrated into the existing topics, learning activities, and teaching delivery methods in the curricula. We also conducted a needs assessment by using a Delphi survey to compare the differences between educators and learners in prioritizing topics for RLO development [19]. After determining the topics and scope of 23 RLOs, we engaged the learners in RLO cocreation, especially in the storyboard development step. We evaluated learners’ knowledge, confidence, and satisfaction of the storyboarding session by using a pre-post survey and explored their perception and experience of the cocreation process qualitatively [20]. We provided RLO specification writing training to the educators who subsequently wrote the content for RLOs. Once RLO specifications were finalized, a content review was conducted before moving on to technical development. A small pilot test was conducted with student champions on RLO usability before full implementation.

### RLO Implementation Phase

The 23 RLOs (Table 1) were made available through the open-access ACo RD repository website [21], Health e-Learning and Media -Open under the University of Nottingham [22], Multimedia Educational Resource for Learning and Online Teaching [23], and the institutional online learning portal and curriculum guidebook.

The focus was on the actual integration of RLO resources into the health sciences curricula. At Universiti Malaya, RLOs were incorporated into various block postings for medicine and an undergraduate semester course for nursing. In Universiti Putra Malaysia, RLOs were used in a professionalism module block, and at Taylor’s University, they were embedded as part of semester courses in pharmacy and biomedical science. Various strategies were implemented to improve the usage of RLOs, including identifying early adopters (department and educators) to use RLOs in teaching, integrating RLOs into the existing curricula, sending reminders to educators, receiving feedback from students, establishing an effective dissemination team, monitoring RLO performance and feedback to educators, and periodic re-examin ing of the implementation strategies.

**Table 1.** The list of reusable learning objects developed for undergraduate students in health care professions.

University, course		RLO^a^ titles (n = 23)
Universiti Malaya, Kuala Lumpur, Malaysia		
Medicine		RLO 1: Prescription Writing—Back to Basics RLO 2: Treatment of A cute I llness RLO 3: Principles of F amily M edicine RLO 4: Factors Affecting Nutrition in the Older Person RLO 5: Growth Faltering in Children RLO 6: Identify C hallenging B ehavior in Health Care Settings RLO 7: How to C onduct a Literature Search
Universiti Putra Malaysia, Selangor, Malaysia		
Medicine		RLO 8: Confidentiality RLO 9: Breaking Bad News RLO 10: Doctor-Patient Relationship RLO 11: Consent RLO 12: Verbal and Non verbal Skills RLO 13: Counseling Skills RLO 14: Ethical Reasoning RLO 15: Social Media Professionalism
Taylor’s University, Selangor, Malaysia		
Pharmacy		RLO 16: Using Nicotine Gum for Smoking Cessation RLO 17: Using Nicotine Patches for Smoking Cessation RLO 18: Using Varenicline for Smoking Cessation
Biomedical science		RLO 19: Body Metabolism RLO 20: DNA Repair RLO 21: DNA Replication RLO 22: Cardiac Output RLO 23: Nervous Regulation of the Heart

a RLO: reusable learning object.

### Evaluation Phase

This study was performed across 3 Malaysian institutions (Universiti Malaya, Universiti Putra Malaysia, and Taylor’s University). Participants in this evaluation were users who used RLOs during the implementation period from May 1, 2020, to February 24, 2022. We used a universal sampling method for data collection.

**Figure 2.**
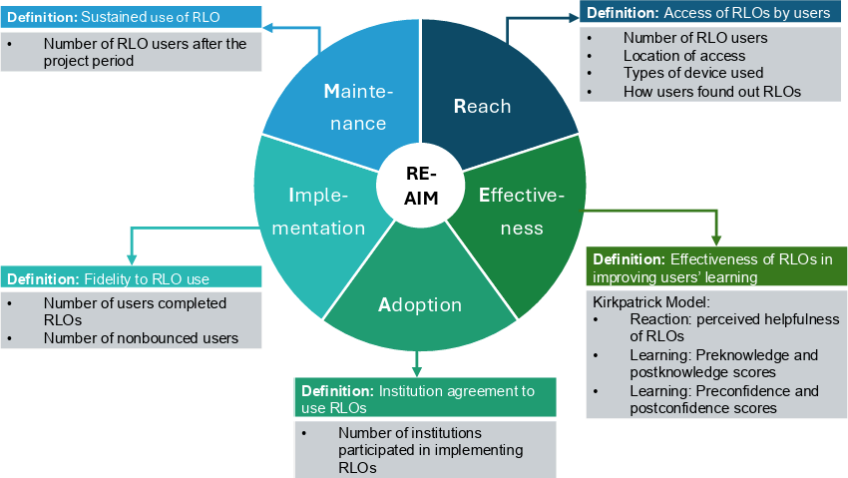
Definitions and outcomes measures of the RE-AIM framework. RE-AIM: Reach, Effectiveness, Adoption, Implementation, and Maintenance; RLO: reusable learning object.

We evaluated the implementation of RLOs by using the RE-AIM framework (Figure 2). The reach domain is defined as the access of RLOs by the users. We assessed reach by measuring the absolute number of RLO users, location of access, types of devices used by the users, and how users found out about our RLOs.

For the effectiveness domain, we applied the 4-level Kirkpatrick model: Reaction, Learning, Behavior, and Results [24]. It is a model that is commonly used in medical education to evaluate the effectiveness of a training program [25]. Reaction was assessed by gauging learners’ perceived helpfulness of RLOs, and learning was measured using pre-RLO and post-RLO knowledge and confidence scores. Behavior measures whether learners are applying the newly acquired knowledge from RLOs in clinical practice, while results measure the ultimate impact of training using RLOs, which includes impact at an organizational level. We did not measure the behavior and results domains in this study due to limitations in the study design.

Adoption refers to the number of institutions that agreed to use RLOs in teaching and learning activities. We captured information on the institutions, departments, and existing modules in which RLOs were implemented. The implementation domain refers to users’ fidelity in using RLOs for their learning. We measured the number of users who completed RLOs and the number of nonbounced users. Nonbounced users are those who are engaged with the site either by clicking on links, visiting multiple pages, or triggering events that indicate interaction, while bounced users are those who visited the RLO web page but only viewed a single page without interacting further before exiting.

The maintenance domain refers to the extent to which the sustained use of RLOs becomes institutionalized or part of the routine teaching and learning practice. We measured the number of users who visited the RLO web page 4 months after the project ended.

### Research Instruments and Data Collection

We used 2 instruments to capture the RLO implementation outcomes: questionnaires and Google Analytics (Table 2). The questionnaires were designed to gather user feedback and usage patterns. We used 2 types of questionnaires: (1) a web-based questionnaire was administered at the completion of each RLO to assess users’ perception of its usefulness (using a 4-point Likert scale: 1-very unhelpful, 2-unhelpful, 3-helpful, and 4-very helpful) and how they discovered RLOs , and (2) a confidence Likert scale and RLO-specific knowledge questionnaire were administered before and immediately after RLO usage. For the knowledge score, RLOs 16, 17, and 18 (using nicotine gum, nicotine patches, and varenicline for smoking cessation) were assessed using 1 set of knowledge questions, while RLOs 20 and 21 (DNA repair and replication) were assessed using another set of knowledge questions relat ed to specific RLO topics. We used GAMM1-Google Analytics in Universiti Malaya and Universiti Putra Malaysia and Moodle monitoring in Taylor’s University to capture the patterns of access (location, number of users, number of completing users).

**Table 2.** RE-AIM (Reach, Effectiveness, Adoption, Implementation, and Maintenance) outcome measures and assessment method s.

RE-AIM measures	Assessment methods
Reach	
Number of RLO^a^ users	Google Analytics
Location of access	Google Analytics
Types of devices used	Google Analytics
How do users find out RLOs	Questionnaire: multiple-choice
Effectiveness	
Perceived helpfulness of RLOs in learning (Kirkpatrick level 1 : reaction)^b^	Questionnaire: 4-point Likert scale
Knowledge and confidence (Kirkpatrick level 2 : learning)	Questionnaire 1: pre- and post-RLO knowledge score; Questionnaire 2: pre- and post-RLO confidence score
Adoption	
Number of institutions who adopted RLOs in teaching and learning	N/A^c^
Implementation	
Number of users who completed RLOs	Google Analytics
Number of nonbounced users^d^	Google Analytics
Maintenance	
Number of RLO accesses 4 months after the ACoRD^e^ project implementation (from February 24 to June 22, 2022)	Google Analytics

a RLO: reusable learning object.

b Perceived helpfulness is measured using a 4-point Likert scale (1-very unhelpful, 2-unhelpful, 3-helpful, and 4-very helpful).

c N/A: not applicable.

d Nonbounced users are defined as users who viewed more than one page.

e ACoRD: Advancing Co-creation of Reusable Learning Objects to Digitise Healthcare Curriculum.

### Data Analysis

For the questionnaire items, categorical data were reported descriptively using proportion and percentage. The mean difference in the pre-RLO and post-RLO scores on knowledge and confidence were analyzed using Mann -Whitney *U* test, as the data were skewed. Statistical analysis was considered significant when *P* < .05. Google Analytics data were reported descriptively using proportion and percentage. All the data were analyzed using SPSS (version 27; IBM Corp) and Microsoft Excel.

### Ethics Approval

Ethic s approval was granted by the Universit i Malaya Research Ethics Committee ( reference UM.TNC2/UMREC-997). All participants provided their written consent. All participant information has been anonymized and deidentified . There was no financial compensation provided to the participants.

## Results

### Overview

**Figure 3.**
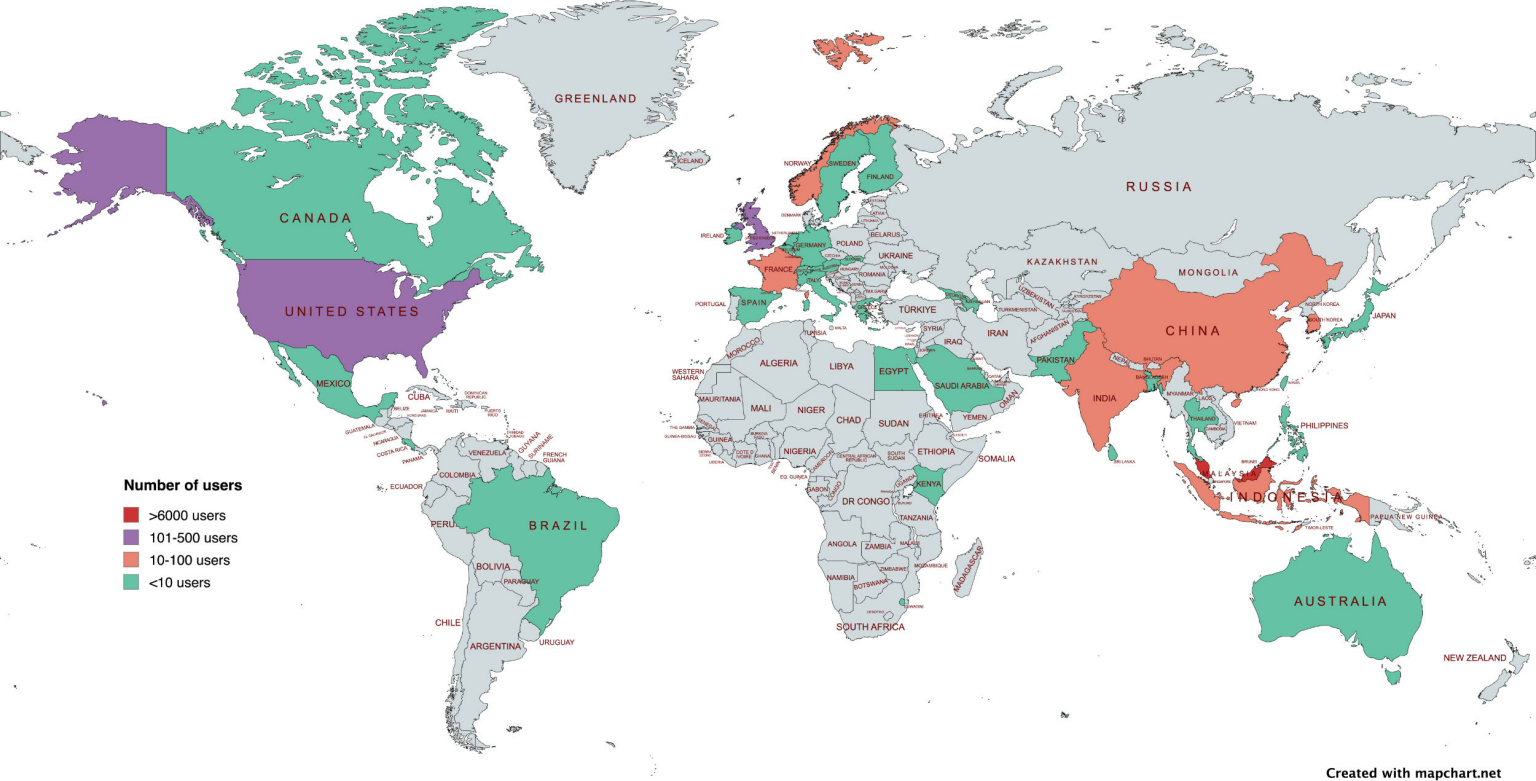
Geographical distribution of the reusable learning object users from 48 countries. There was no country with users between 500 and 6000.

From the launch of the first RLO on May 1, 2020, until February 24, 2022, Google Analytics recorded a cumulative 7622 users from 48 countries (Figure 3). We have reported the results according to the RE-AIM framework.

### Reach

Among the 7622 global users, the majority (n= 6817, 89.4% ) were from Malaysia, while 10.6 % (n= 805) were from other countries. Desktop computers were the predominant devices used to access RLOs ( 6045/7622 , 79.3% ); 20.7 % ( 1577/7622 ) used portable devices such as mobile phones or tablets. The number of users for each RLO varied from 92 to 1014 ( Table 3). The RLOs attracting the highest number of users were RLO 7 (How to C onduct L iterature R esearch , n = 1014), RLO 1 (Prescription W riting : B ack to B asics , n = 530), and RLO 17 (Using N icotine P atches for Smoking Cessation , n = 511). RLOs with fewer than 100 users were RLO 10 (Doctor-Patient Relationship ) and RLO 23 (Nervous R egulation of the H eart ).

Out of 2118 respondents to the questionnaire, the majority used RLOs because they were part of the course learning resource (n= 1901, 89.8% ), while others received a recommend ation from peers, colleagues, or lecturers (n= 87, 8.8% ). A small proportion of the respondents ( 30/2118, 1.5% ) found RLOs through the Health e-Learning and Media open database, open educational catalogs, or general internet searches.

**Table 3.** Number of users for each reusable learning object during project implementation.

RLO^a^ type	Users, n
RL O 1	5 30
RL O 2	3 66
RL O 3	4 67
RL O 4	3 10
RL O 5	3 63
RL O 6	184
RL O 7	10 14
RL O 8	2 34
RL O 9	187
RLO 10	97
RLO 11	2 12
RLO 12	3 36
RLO 13	187
RLO 14	3 35
RLO 15	388
RLO 16	4 32
RLO 17	5 11
RL O 18	418
RLO 19	191
RLO 20	3 65
RLO 21	179
RLO 22	2 24
RLO 23	92

a RLO: reusable learning object.

### Effectiveness

**Figure 4.**
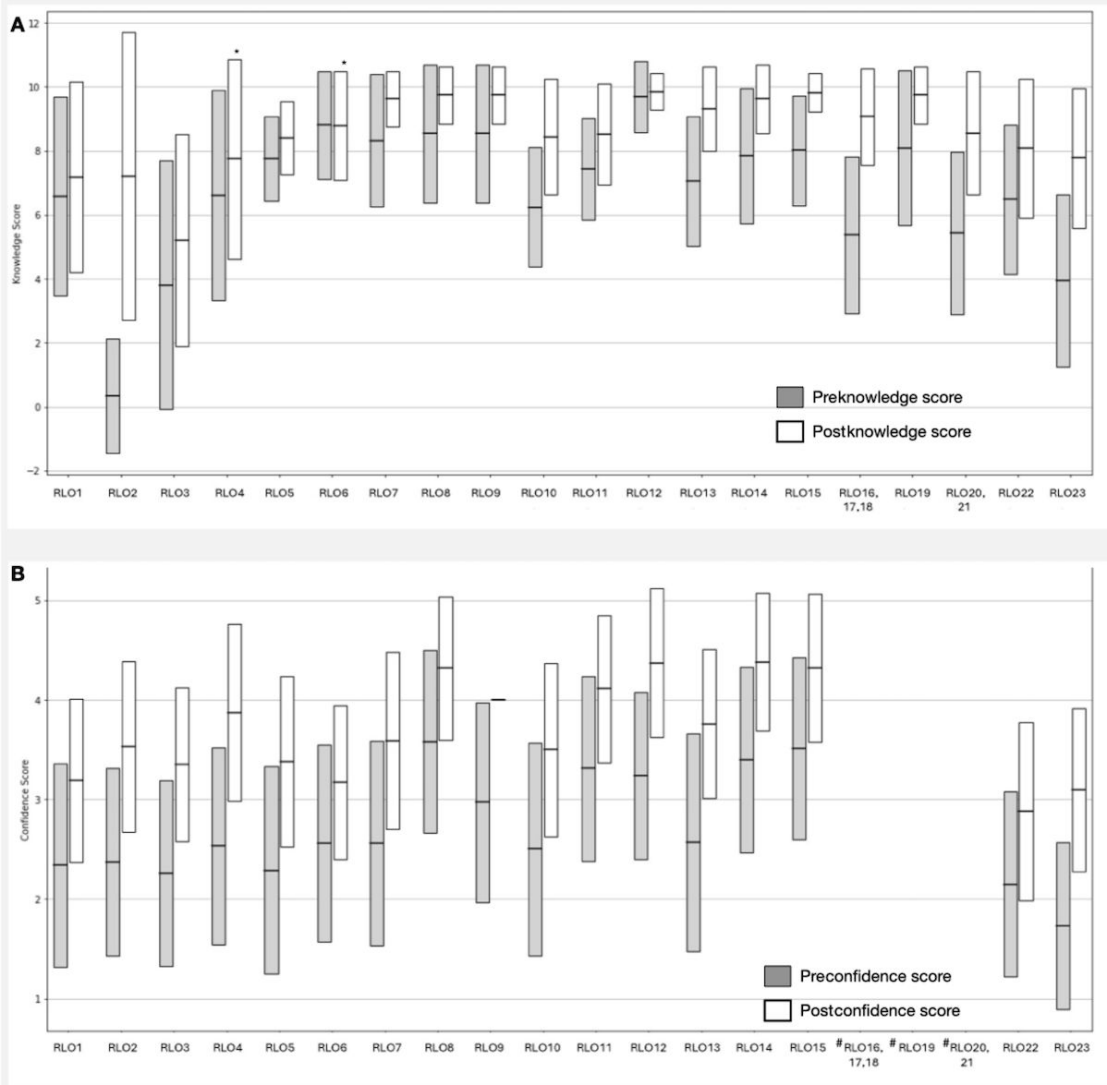
( **A** ) Comparison of pre knowledge and postknowledge scores for each reusable learning object. ( **B** ) Comparison of pre confidence and postconfidence scores for each reusable learning object. * *P* > .05 . # indicates missing data . RLO: reusable learning object.

Most users rated RLOs as very helpful ( 1452 / 2071, 70.1% ) and helpful ( 601/ 2071, 29% ). We evaluated the pre knowledge and postknowledge scores across all RLOs. The mean preknowledge score ranged from 0.33 to 9.6, while the postknowledge score ranged from 5.20 to 9.85. A significant improvement in the knowledge scores was reported for all RLOs after their use ( *P* < .05) (Figure 4), except for RLO 4 and RLO 6, which had high mean baseline preknowledge scores of 6.61 and 8.80, respectively (Table S1 in Multimedia Appendix 1 ). Additionally, we assessed the confidence scores for 17 RLOs (excluding 5 RLOs, ie, RLOs 16 -21 due to missing data) and found a significant improvement in the mean confidence scores after utilization for all 17 RLOs ( *P* < .05) (Table S2 in Multimedia Appendix 1 ).

### Adoption

All RLOs have been adopted by the 3 Malaysian universities ( Universiti Malaya, Universiti Putra Malaysia, and Taylor’s University). At Universiti Malaya, the departments of primary care medicine, medicine (geriatrics), pediatrics , and nursing have incorporated RLOs into their teaching and learning curricula. Universiti Putra Malaysia has used RLOs within its professional development module, while Taylor’s University has implemented them in the pharmacy and biomedical undergraduate courses.

### Implementation

The completion rates for RLOs varied widely, with percentages ranging from 5.6% (10/179) to 8 5% (78/92) as shown in Table 4. Only RLO 8 (Confidentiality), RLO 15 (Social Media Professionalism), and RLO 23 (Nervous Regulation of the Heart) had a completion rate exceeding 50 %. The proportion of nonbounced users, defined as users who viewed more than one page, ranged from 16.3% (165/1014) to 8 8.5% (370/418) . Notably, data on nonbounced users for RLOs 8 -15 from Universiti Putra Malaysia were unavailable due to the absence of a tracking function on each RLO page in Google Analytics ( because of technical errors).

**Table 4.** Number of users who completed reusable learning objects and number of nonbounced users during the implementation period.

RLO^a^ type	Accesses, n	Users who completed RLOs, n (%)	Nonbounced^b^ users, n (%)
RLO 1	530	205 (38.7)	129 (24.3)
RLO 2	366	90 (24.6)	97 (26.5)
RLO 3	467	116 (24.8)	143 (30.6)
RLO 4	310	46 (14.8)	64 (20.7)
RLO 5	363	53 (14.6)	71 (19.6)
RLO 6	184	33 (17.9)	49 (26.6)
RLO 7	1014	75 (7.4)	165 (16.3)
RLO 8	234	170 (72.7)	—^c^
RLO 9	187	68 (36.4)	—
RLO 10	97	35 (36)	—
RLO 11	212	100 (47.2)	—
RLO 12	336	164 (48.8)	—
RLO 13	187	60 (32.1)	—
RLO 14	335	116 (34.6)	—
RLO 15	388	204 (52.6)	—
RLO 16	432	121 (28)	352 (81.5)
RLO 17	511	113 (22.1)	351 (68.7)
RLO 18	418	109 (26.1)	370 (88.5)
RLO 19	191	23 (12)	68 (35.6)
RLO 20	365	61 (16.7)	271 (74.3)
RLO 21	179	10 (5.6)	111 (62)
RLO 22	224	76 (33.9)	147 (65.6)
RLO 23	92	78 (84.8)	30 (32.6)

a RLO: reusable learning object.

b Users who viewed more than one page.

c Not available.

### Maintenance

**Figure 5.**
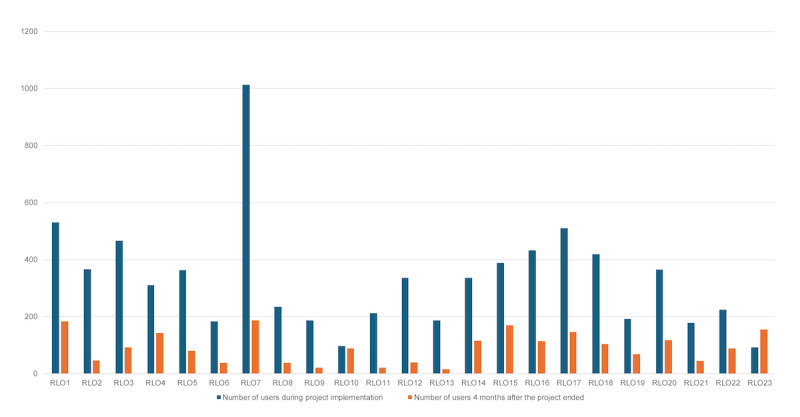
Number of users who accessed reusable learning objects 4 months after the end of the project *.*

After the ACoRD project ended on February 24, 2022, a total of 2107 users continued to access RLOs in the subsequent 4 months, ranging from 15 to 187 accesses per RLO ( Figure 5 ).

## Discussion

### Principal Findings

This study reports the successful implementation of RLOs in health care education across 3 Malaysian higher educational institutions by using the RE-AIM framework. Our findings offer a broad perspective on how the impact and outcomes of e- learning objects can be measured and evaluated systematically to inform e- learning implementation strategies.

Our study shows that RLOs were accessed by a more diverse group of learners than initially anticipated, extending beyond the Malaysian institutions, where RLOs were intended to be used. Leveraging on the unique characteristics of reusability in RLOs [26], we adopted an open educational resource approach in their deployment. This strategy significantly broadened the reach of RLOs, making them available to learners across different disciplines. Consequently, RLOs addressing more general subjects such as literature review, prescription writing, and smoking cessation attracted a larger user base compared to those focusing on discipline-specific topics.

Throughout the ACoRD project, pragmatic implementation and dissemination strategies were employed to engage a broader audience. These strategies encompassed identifying early adopters and educator champions , sending of reminders to educators about incorporating RLOs in their teaching , monitoring of RLO performance at regular intervals , and providing feedback to educators. A study on mobile health e- learning courses highlighted the advantages of iterative feedback among developers, early adopters, and end users, which aid in the refinement of existing e- learning implementation strategies and the development of new ones [27]. The ACoRD project featured a dedicated dissemination team that organized and executed activities to promote RLOs through conferences and engagement workshops, extending beyond our institutions. Establishing collaborative partnerships with other higher educational institutions is an important strategy to boost visibility and ensure the long-term use of e- learning resources [28].

In our study, users perceived RLOs as beneficial for their learning, with most RLOs demonstrating an improvement in users’ knowledge and confidence levels. These findings are aligned with previous studies that have reported improvements in knowledge and understanding following RLO usage [16,29,30]. However, evaluating the effectiveness of e- learning resources remains a challenge. Most studies measured learners’ reactions and learning s such as knowledge, usefulness, confidence, satisfaction, and motivation [31]. Some studies examining the effectiveness of RLOs demonstrated behavior change in relation to prescribing behavior and hearing aid use [32,33]. Measuring higher levels of learning such as behavior change and impact of learning is complex due to the time and cost involved [34]. We propose that the evaluation of RLO effectiveness should go beyond individual learning outcomes to assess their impact within the wider teaching and learning ecosystem. This could include evaluating RLOs’ effectiveness from educators’ perspectives and examining how RLOs contribute to student empowerment in self-directed learning [6].

In the adoption domain of RE-AIM, RLOs developed by the ACoRD project were adopted by all 3 Malaysian institutions. Our study underscore s the significance of planning and initiating implementation strategies during the development phase (preimplementation) with stakeholders and institutional engagement. Engaging institutional faculty, students, and stakeholders is critical for the successful adoption of e- learning [35]. The cocreation process and involvement of stakeholders throughout the ASPIRE process facilitates the feeling of ownership of the materials produced, thereby leading to use and reuse. To ensure the widespread adoption of e- learning innovation, it is crucial to engage with and consider the perspectives of diverse stakeholders, as the implementation of e- learning necessitates changes in teaching, learning, management, and infrastructure within the institution [36].

In our study, although RLOs reached a significant number of users, the proportion of users who completed RLOs (5.6% ‐ 84.4 %) and nonbounced users (16.3% ‐ 81.5 %) was relatively low for most RLOs. Chen et al [ 37 ] reported a similar finding, with a low nonbounced rate of 40 % for an undergraduate online course . The wide discrepancy in the completion and nonbounced rates among RLOs may be attributed to the different levels of learning across various learner groups. RLOs integrated into the curriculum as teaching and learning materials achieved a completion rate greater than 70 % (RLO 8 and RLO 23), suggesting that learners are more invested in completing these RLOs. Conversely, RLO 7 attracted the highest number of users (n = 1014) ; yet, its completion rate was only 7.4 % ( 75/1014 ). Our findings show that Google Analytics is beneficial for examining the fidelity of RLO usage, as it provides insights into learners’ behaviors. Assessing the fidelity of RLO usage is crucial for educators and institutions to gauge the effectiveness of RLOs in teaching and learning [38].

Users continued to access RLOs after the conclusion of the ACoRD project, which indicates the sustainability of RLOs. The main strategies that we identified were the integration of RLOs into the existing health care curriculum and developing RLOs by using a robust cocreation methodology. Identifying the learning needs of teachers and learners prior to the development of RLOs facilitated their integration into the curriculum [19]. We also implemented the educator-as-champion strategy, which involves educators in content development and subsequently utilizing RLOs in their teaching. e-Learning champions among academicians in higher educational institutions are the key players in fostering the integration of technology into teaching and learning [39].

### Limitations

Our study has several limitations. Since each RLO was developed and launched at different times, discrepancies in the implementation periods made data interpretation challenging. We used Google Analytics to capture the reach of our RLOs. However, we could not confirm whether access came from unique users or unique IP addresses. There is a possibility that some users could have a ccessed RLOs multiple times by using different IP addresses. As this study employ s a pragmatic approach to capture evaluation outcomes in real-time, missing data were inevitable; however, it did not substantially impact the overall findings. Strategies were implemented pragmatically throughout the project, with practical consideration and changes made to address implementation issues; as a result, we were unable to identify and measure the effectiveness of each implementation strategy.

### Conclusion

We employed the RE-AIM framework to systematically evaluate the implementation success of e- learning resources, identifying gaps and strategies for improvement. This study highlight s the development, implementation process, and implementation outcome indicators of open-access RLOs in health care education. To enhance future e- learning implementation efforts, we recommend incorporating the RE-AIM framework into outcome evaluations to provide a more comprehensive evaluation of e- learning implementation.

## Supplementary material

10.2196/63882 Multimedia Appendix 1 Pre knowledge and postknowledge and confidence scores for each reusable learning object.

## A bbreviations

ACoRD: Advancing Co-creation of Reusable Learning Objects to Digitise Healthcare Curriculum
ASPIRE: Aim, Storyboarding, Populating, Implementing, Release, and Evaluation
RE-AIM: Reach, Effectiveness, Adoption, Implementation, and Maintenance
RLO: reusable learning object

## References

[1] Wentling TL, Waight C, Gallaher J, La Fleur J, Wang C, Kanfer A (2000). E-learning: a review of literature. ResearchGate.

[2] Wang ZY, Zhang LJ, Liu YH (2021). The effectiveness of e
-learning in continuing medical education for tuberculosis health workers: a quasi-experiment from China. Infect Dis Poverty.

[3] Sadeghi R, Sedaghat MM, Sha Ahmadi F (2014). Comparison of the effect of lecture and blended teaching methods on students’ learning and satisfaction. J Adv Med Educ Prof.

[4] Ruiz JG, Mintzer MJ, Leipzig RM (2006). The impact of e
-learning in medical education. Acad Med.

[5] Naveed QN, Qureshi MRN, Tairan N (2020). Evaluating critical success factors in implementing e
-learning system using multi-criteria decision-making. PLoS One.

[6] Barteit S, Guzek D, Jahn A, Bärnighausen T, Jorge MM, Neuhann F (2020). Evaluation of e-learning for medical education in low- and middle-income countries: a
systematic review. Comput Educ.

[7] Proctor E, Silmere H, Raghavan R (2011). Outcomes for implementation research: conceptual distinctions, measurement challenges, and research agenda. Adm Policy Ment Health.

[8] Palmerola ED (2024). Clarification evaluation of e-learning implementation: a developmental research design. Am J Educ Technol.

[9] Kirkpatrick D, Kirpatrick JD (2011). Evaluating t
raining p
rograms: t
he f
our l
evels. American J Eval.

[10] Stufflebeam D (2002). Evaluation Models.

[11] Gandomkar R (2018). Comparing Kirkpatrick’s original and new model with CIPP evaluation model. J Adv Med Educ Prof.

[12] Peterson C (2003). Bringing ADDIE to life: instructional design at its best. ERIC.

[13] Holtrop JS, Estabrooks PA, Gaglio B (2021). Understanding and applying the RE-AIM framework: clarifications and resources. J Clin Transl Sci.

[14] Gisondi MA, Keyes T, Zucker S, Bumgardner D (2023). Teaching LGBTQ+ health, a web-based faculty development course: program evaluation study using the RE-AIM framework. JMIR Med Educ.

[15] Golden RE, Sanders AM, Frayne SM (2023). RE-AIM applied to a primary care workforce training for rural providers and nurses: the Department of Veterans Affairs’ Rural Women’s Health Mini-Residency. Front Health Serv.

[16] Bath-Hextall F, Wharrad H, Leonardi-Bee J (2011). Teaching tools in evidence based practice: evaluation of reusable learning objects (RLOs) for learning about meta-analysis. BMC Med Educ.

[17] Lim HM, Ng CJ, Wharrad H (2022). Knowledge transfer of e-learning objects: lessons learned from an intercontinental capacity building project. PLoS ONE.

[18] Wharrad H, Windle R, Taylor M, Konstantinidis ST, Bamidis PD, Zary N (2021). Digital Innovations in Healthcare Education and Training.

[19] Lim HM, Ng CJ, Teo CH (2021). Prioritising topics for developing e-learning resources in healthcare curricula: a
comparison between students and educators using a modified Delphi survey. PLoS ONE.

[20] Lim HM, Teo CH, Hong WH, Lee YK, Lee PY, Ng CJ (2024). Empowering students in co-creating e-learning resources through a virtual hackathon. TAPS.

[21] (2018). The ACoRD Project.

[22] HELM Open. University of Nottingham.

[23] MERLOT multimedia educational resource for learning and online teaching. MERLOT.

[24] Kirkpatrick DL, Brown SM, Seidner CJ (1998). Evaluating Corporate Training: Models and Issues.

[25] Ragsdale JW, Berry A, Gibson JW (2020). Evaluating the effectiveness of undergraduate clinical education programs. Med Educ Online.

[26] Windle RJ, Wharrad H, McCormick D, Laverty H, Taylor MG (2010). Sharing and reuse in OER: experiences gained from open reusable learning objects in health. JIME.

[27] Philpot LM, Ahrens DJ, Eastman RJ (2023). Implementation of e-learning solutions for patients with chronic pain conditions. Digit Health.

[28] Gallagher S, Murphy P (2024). Implementing inter-institutional lifelong sustainability education: the UNI-ECO e-learning case study. Ir J Acad Pract.

[29] Hardie P, Donnelly P, Greene E (2021). The application of reusable learning objects (RLOs) in preparation for a simulation laboratory in medication management: an evaluative study. Teaching Learning Nurs.

[30] Redmond C, Davies C, Cornally D (2018). Using reusable learning objects (RLOs) in wound care education: u
ndergraduate student nurse’s evaluation of their learning gain. Nurse Educ Today.

[31] de Leeuw R, de Soet A, van der Horst S, Walsh K, Westerman M, Scheele F (2019). How we evaluate postgraduate medical e-learning: systematic review. JMIR Med Educ.

[32] Lymn JS, Bath-Hextall F, Wharrad HJ (2008). Pharmacology education for nurse prescribing students - a lesson in reusable learning objects. BMC Nurs.

[33] Ferguson M, Brandreth M, Brassington W, Leighton P, Wharrad H (2016). A randomized controlled trial to evaluate the benefits of a multimedia educational program for first-time hearing aid users. Ear Hear.

[34] El Nsouli D, Nelson D, Nsouli L (2023). The application of Kirkpatrick’s evaluation model
in the assessment of interprofessional simulation activities involving pharmacy students: a systematic review. Am J Pharm Educ.

[35] Vovides Y, Chale SB, Gadhula R (2014). A systems approach to implementation of e-learning in medical education: five MEPI schools’ journeys. Acad Med.

[36] de Souza Rodrigues MA, Chimenti P, Nogueira ARR (2021). An exploration of e-learning adoption in the educational ecosystem. Educ Inf Technol.

[37] Chen Y, Deng X, Huang Q, Luo H Patterns and trends in online learning behaviors: evidence from Google analytics.

[38] Mudawi NA, Pervaiz M, Alabduallah BI (2023). Predictive analytics for sustainable e-learning: tracking student behaviors. Sustainability.

[39] Gachago D, Morkel J, Hitge L, van Zyl I, Ivala E (2017). Developing e-learning champions: a design thinking approach. Int J Educ Technol High Educ.

