# Supplementary material for: Implementation Outcomes of Reusable Learning Objects in Health Care Education Across Three Malaysian Universities: Evaluation Using the RE-AIM Framework
Source: JMIR Med Educ. 2025 Jul 23;11:e63882. doi: 10.2196/63882 (PMC12519310; doi:10.2196/63882)
Supplement: Multimedia Appendix 1 [file mededu-v11-e63882-s001.docx]

**Multimedia Appendix 1**

Pre- and post-knowledge and confidence scores for each RLO

**Table S1: Pre- and post-knowledge scores for each RLO**

| RLO | No of Questions | Pre | | | Post | | | | Post 6 weeks | | | |
| --- | --- | --- | --- | --- | --- | --- | --- | --- | --- | --- | --- | --- |
|  |  | N | Mean | SD | N | Mean | SD | P- value | N | Mean | SD | P- value |
| **University of Malaya** | | | | | | | | | | | | |
| LO 1: Prescription Writing: back to basic | 2 | 300 | 6.58 | 3.10 | 268 | 7.18 | 2.97 | 0.019 | 71 | 7.75 | 2.78 | 0.004 |
| RLO 2: Treatment of acute illness | 10 | 122 | 0.33 | 1.79 | 140 | 7.21 | 4.50 | <0.001 | 23 | 1.30 | 3.44 | 0.197 |
| RLO 3: Principles of family medicine | 2 | 185 | 3.81 | 3.89 | 205 | 5.20 | 3.32 | <0.001 | 21 | 8.10 | 3.70 | <0.001 |
| RLO 4: Factors Affecting Nutrition in the Older Person | 2 | 87 | 6.61 | 3.28 | 31 | 7.74 | 3.12 | 0.097 | 1 | 10.0 | - | - |
| RLO 5: Growth Faltering in Children | 10 | 80 | 7.75 | 1.32 | 64 | 8.39 | 1.14 | 0.002 | 5 | 7.80 | 1.10 | 0.934 |
| RLO 6: Identify challenging behavior in healthcare settings | 3 | 89 | 8.80 | 1.69 | 71 | 8.78 | 1.71 | 0.935 | 4 | 10.0 | 0 | <0.001 |
| RLO 7: How to conduct a literature search | 6 | 262 | 8.32 | 2.06 | 160 | 9.62 | 0.86 | <0.001 | 17 | 8.53 | 1.85 | 0.685 |
| **University Putra Malaysia** | | | | | | | | | | | | |
| RLO 8: Confidentiality | 5 | 96 | 8.54 | 2,15 | 91 | 9.74 | 0.89 | <0.001 | 17 | 9.41 | 1.3 | 0.11 |
| RLO 9: Breaking bad news | 3 | 96 | 8.54 | 2.15 | 91 | 9.74 | 0.89 | <0.001 | 17 | 9.41 | 1.3 | 0.11 |
| RLO 10: Doctor-patient relationship | 6 | 75 | 6.24 | 1.86 | 73 | 8.44 | 1.81 | <0.001 | 3 | 6.11 | 0.96 | 0.904 |
| RLO 11: Consent | 5 | 162 | 7.44 | 1.58 | 184 | 8.52 | 1.59 | <0.001 | 5 | 9.50 | 1.00 | 0.011 |
| RLO 12: Verbal and non-verbal skills | 5 | 236 | 9.68 | 1.11 | 211 | 9.85 | 0.57 | 0.045 | 1 | 10.00 | - | 0.772 |
| RLO 13: Counselling skills | 5 | 94 | 7.06 | 2.02 | 88 | 9.31 | 1.31 | <0.001 | 20 | 8.4 | 1.39 | 0.006* |
| RLO 14: Ethical reasoning | 5 | 165 | 7.84 | 2.11 | 254 | 9.62 | 1.08 | <0.001 | 3 | 6.66 | 1.15 | 0.34 |
| RLO 15: Social media | 5 | 277 | 8.01 | 1.71 | 242 | 9.82 | 0.60 | <0.001 | 2 | 9.00 | 1.41 | 0.417 |
| **Taylors’ University** | | | | | | | | | | | | |
| RLO 16: Using Nicotine Gum for Smoking Cessation | 6 | 104 | 5.37 | 2.44 | 87 | 9.06 | 1.50 | <0.001 | 90 | 8.07 | 2.04 | <0.001* |
| RLO 17: Using Nicotine Patches for Smoking Cessation |  |  |  |  |  |  |  |  |  |  |  |  |
| RLO 18: Using Varenicline for Smoking Cessation |  |  |  |  |  |  |  |  |  |  |  |  |
| RLO 19: Body metabolism | 3 | 66 | 8.08 | 2.42 | 52 | 9.74 | 0.9 | <0.001 | 30 | 8.78 | 2.39 | 0.192 |
| RLO 20: DNA repair | 5 | 68 | 5.43 | 2.54 | 68 | 8.56 | 1.91 | <0.001 | 19 | 8.21 | 2.57 | <0.001 |
| RLO 21: DNA replication | Not available | | | | | | | | | | | |
| RLO 22: Cardiac Output | 4 | 62 | 6.49 | 2.33 | 60 | 8.08 | 2.18 | <0.001 | 52 | 7.74 | 1.93 | 0.003 |
| RLO 23: Nervous regulation of the heart | 4 | 62 | 3.95 | 2.70 | 64 | 7.77 | 2.19 | <0.001 | 54 | 6.30 | 2.60 | <0.001 |

**Table S2. Pre- and post-RLO confidence scores for each RLO**

| RLO | Pre | | | Post | | | | Post 6 weeks | | | |
| --- | --- | --- | --- | --- | --- | --- | --- | --- | --- | --- | --- |
|  | N | Mean | SD | N | Mean | SD | P- value | N | Mean | SD | P- value |
| **University of Malaya** | | | | | | | | | | | |
| RLO 1: Prescription Writing: back to basic | 164 | 2.34 | 1.02 | 143 | 3.19 | 0.82 | <0.001 | 58 | 3.14 | 0.81 | <0.001 |
| RLO 2: Treatment of acute illness | 81 | 2.37 | 0.94 | 97 | 3.53 | 0.86 | <0.001 | 20 | 2.90 | 0.79 | 0.011 |
| RLO 3: Principles of family medicine | 139 | 2.26 | 0.93 | 158 | 3.35 | 0.77 | <0.001 | 18 | 3.00 | 0.77 | 0.001 |
| RLO 4: Factors Affecting Nutrition in the Older Person | 75 | 2.53 | 0.99 | 31 | 3.87 | 0.89 | <0.001 | 1 | 3 | - | - |
| RLO 5: Growth Faltering in Children | 31 | 2.29 | 1.04 | 29 | 3.38 | 0.86 | <0.001 | 2 | 3.00 | 0 | 0.001 |
| RLO 6: Identify challenging behavior in healthcare settings | 86 | 2.56 | 0.99 | 71 | 3.17 | 0.77 | <0.001 | 4 | 3.25 | 0.96 | 0.175 |
| RLO 7: How to conduct a literature search | 191 | 2.56 | 1.03 | 116 | 3.59 | 0.89 | <0.001 | 14 | 2.64 | 1.08 | 0.774 |
| **Universiti Putra Malaysia** | | | | | | | | | | | |
| RLO 8: Confidentiality | 180 | 3.58 | 0.92 | 180 | 4.32 | 0.72 | <0.001 | 5 | 4.6 | 0.55 | 0.015 |
| RLO 9: Breaking Bad News | 68 | 2.97 | 1.00 | 78 | 4.00 | 0.00 | <0.001 | 17 | 3.7 | 0.68 | 0.006 |
| RLO 10: Doctor-Patient Relationship | 75 | 2.5 | 1,07 | 73 | 3.5 | 0.87 | <0.001 | 3 | 3.34 | 0.58 | 0.189 |
| RLO 11: Consent | 162 | 3.31 | 0.93 | 184 | 4.11 | 0.74 | <0.001 | 4 | 4.00 | 0.00 | 0.142 |
| RLO 12: Verbal and Non-verbal | 122 | 3.24 | 0.84 | 211 | 4.37 | 0.75 | <0.001* | 1 | 5.00 | - | 0.040 |
| RLO 13: Counselling Skills | 68 | 2.57 | 1.09 | 68 | 3.76 | 0.75 | <0.001 | 20 | 3.6 | 0.68 | <0.0011 |
| RLO 14: Ethical Reasoning | 165 | 3.4 | 0/93 | 254 | 4.38 | 0.69 | <0.001 | 3 | 3.67 | 0.58 | 0.622 |
| RLO 15: Social Media Professionalism | 159 | 3.51 | 0.91 | 241 | 4.32 | 0.74 | <0.001 | 2 | 5.00 | 0.00 | 0.023 |
| Taylor’s University | | | | | | | | | | | |
| RLO 16: Using Nicotine Gum for Smoking Cessation | Not available | | | | | | | | | | |
| RLO 17: Using Nicotine Patches for Smoking Cessation | Not available | | | | | | | | | | |
| RLO 18: Using Varenicline for Smoking Cessation | Not available | | | | | | | | | | |
| RLO 19: Body metabolism | Not available | | | | | | | | | | |
| RLO 20: DNA repair | Not available | | | | | | | | | | |
| RLO 21: DNA replication | Not available | | | | | | | | | | |
| RLO 22: Cardiac Output | 59 | 2.15 | 0.93 | 60 | 2.88 | 0.89 | <0.001 | 51 | 2.88 | 1.05 | <0.001 |
| RLO 23: Nervous regulation of the heart | 60 | 1.73 | 0.84 | 62 | 3.10 | 0.82 | <0.001 | 53 | 2.81 | 1.00 | <0.001 |
